# A *Glycine soja* group S2 bZIP transcription factor *GsbZIP67* conferred bicarbonate alkaline tolerance in *Medicago sativa*

**DOI:** 10.1186/s12870-018-1466-3

**Published:** 2018-10-13

**Authors:** Shengyang Wu, Pinhui Zhu, Bowei Jia, Junkai Yang, Yang Shen, Xiaoxi Cai, Xiaoli Sun, Yanming Zhu, Mingzhe Sun

**Affiliations:** 1 Plant Bioengineering Laboratory, Northeast Agricultural University, Harbin, 150030 People’s Republic of China; 2 Crop Stress Molecular Biology Laboratory, Heilongjiang Bayi Agricultural University, Daqing, 163319 People’s Republic of China

**Keywords:** Transcription factor, bZIP family, Bicarbonate alkaline stress, Wild soybean, Alfalfa

## Abstract

**Background:**

Even though bicarbonate alkaline stress is a serious threat to crop growth and yields, it attracts much fewer researches than high salinity stress. The basic leucine zipper (bZIP) transcription factors have been well demonstrated to function in diverse abiotic stresses; however, their biological role in alkaline tolerance still remains elusive. In this study, we functionally characterized a bZIP gene from *Glycine soja GsbZIP67* in bicarbonate alkaline stress responses.

**Results:**

*GsbZIP67* was initially identified as a putative bicarbonate responsive gene, on the basis of previous RNA-seq data of 50 mM NaHCO_3_-treated *Glycine soja* roots. GsbZIP67 protein possessed a conserved bZIP domain, and belonged to the group S2 bZIP, which is yet less well-studied. Our studies showed that GsbZIP67 targeted to nucleus in *Arabidopsis* protoplasts, and displayed transcriptional activation activity in yeast cells. The quantitative real-time PCR analyses unraveled the bicarbonate stress responsive expression and tissue specific expression of *GsbZIP67* in wild soybean. Further phenotypic analysis illustrated that *GsbZIP67* overexpression in alfalfa promoted plant growth under bicarbonate alkaline stress, as evidenced by longer roots and shoots. Furthermore, *GsbZIP67* overexpression also modified the physiological indices of transgenic alfalfa under bicarbonate alkaline stress. In addition, the expression levels of several stress responsive genes were also augmented by *GsbZIP67* overexpression.

**Conclusions:**

Collectively, in this study, we demonstrated that *GsbZIP67* acted as a positive regulator of plant tolerance to bicarbonate alkaline stress. These results provide direct genetic evidence of group S2 bZIPs in bicarbonate alkaline stress, and will facilitate further studies concerning the cis-elements and/or downstream genes targeted by *GsbZIP67* in stress responses.

**Electronic supplementary material:**

The online version of this article (10.1186/s12870-018-1466-3) contains supplementary material, which is available to authorized users.

## Background

Soil salinization-alkalization imposes a direct detrimental effect on plant survival, and is a serious threat to crop yields and agricultural production worldwide [1]. Around the world, approximately 830 million hectares are saline-alkaline soils, which consist of about 53% alkaline soils and 47% saline soils. Compared to saline soils (NaCl/Na_2_SO_4_), alkaline soils are composed primarily of NaHCO_3_/Na_2_CO_3_. An increasing number of researches have suggested that bicarbonate/carbonate alkaline stress imposes much severer repression on plant growth through the complex damages derived from excess Na^+^, HCO_3_^−^/CO_3_^2−^, high-pH (> 8.5) and poor soil structure [2]. Hence, improving the bicarbonate/carbonate alkaline tolerance is of profound importance to crop yields, and will greatly secure the food security. Unfortunately, compared with salt stress, fewer studies have been reported concerning the bicarbonate/carbonate alkaline tolerance.

As direct regulators of gene expression, transcription factors (TFs) influence essentially all aspects of plant growth and development, as well as stress responses. The basic leucine zipper (bZIP) TFs are characterized by a basic region responsible for sequence-specific DNA binding, and a leucine zipper domain required for homo−/hetero-dimerization [3]. There are 75 bZIPs in *Arabidopsis* genome, which are clustered into 10 groups (group A-I, S) [3]. The S group was further divided into group S1, S2 and S3 [4]. Among them, group A bZIPs are well characterized to mediate ABA and stress responses, while group D bZIPs are involved in development and pathogen defense. Group H play a paramount role in promoting photomorphogenesis. Group C/S1 bZIPs could specifically form homo- and heterodimers, and are involved in the low energy and nutrient signaling [5]. Moreover, several studies reported that C/S1 bZIPs also acted in biotic and abiotic stress responses [6, 7]. However, the biological functions of group S2/S3 bZIPs have not been unraveled until now.

Very recently, a total of 160 bZIP genes were identified in soybean (*Glycine max*) genome [8]. Until now, the positive function of several GmbZIPs in abiotic stress responses has been reported, for example *GmbZIP132/110* in salt stress [9, 10], *GmbZIP44/62/78* in salt and freezing stresses [11], as well as *GmbZIP1* in cold, salt and drought stresses [12]. However, the biological role of GmbZIPs in bicarbonate/carbonate alkaline stress still remains elusive.

Compared with cultivated soybean, wild soybean (*Glycine soja*) is much more tolerant to salt-alkaline stress. Previously, from over 300 wild soybean lines, we characterized one line G07256 showing the highest bicarbonate alkaline tolerance, and transcriptionally identified the bicarbonate alkaline responsive genes [13, 14], such as *GsCBRLK* [15, 16], *GsERF71* [17] and *Gshdz4* [18]. Among them, some were transformed into *Medicago savita* to breed transgenic alfalfa with superior bicarbonate alkaline tolerance [19–21]. In this study, we further identified a group S2 bZIP TF, *GsbZIP67*, as a novel bicarbonate stress responsive gene. Our results showed that GsbZIP67 localized in nucleus and displayed transcriptional activation activity. Overexpression of *GsbZIP67* in alfalfa improved the bicarbonate alkaline tolerance and promoted the expression of stress responsive genes. Collectively, these results provide direct genetic evidence of *GsbZIP67* in bicarbonate alkaline tolerance, and will greatly facilitate to further explore the potential cis-elements and/or downstream genes targeted by *GsbZIP67* in the regulation of bicarbonate alkaline tolerance.

## Results

### Sequence and phylogenetic analysis of GsbZIP67

Based on previous wild soybean RNA-seq data [14], Glyma08g28220 encoding a bZIP TF was identified as a putative bicarbonate alkaline stress responsive gene. According to the genome wide identification of *G. max* bZIP family [8], Glyma08g28220 was designated as *GmbZIP67*. Hence, we cloned its orthologues in *G. soja* and named it as *GsbZIP67*.

**Fig. 1:**
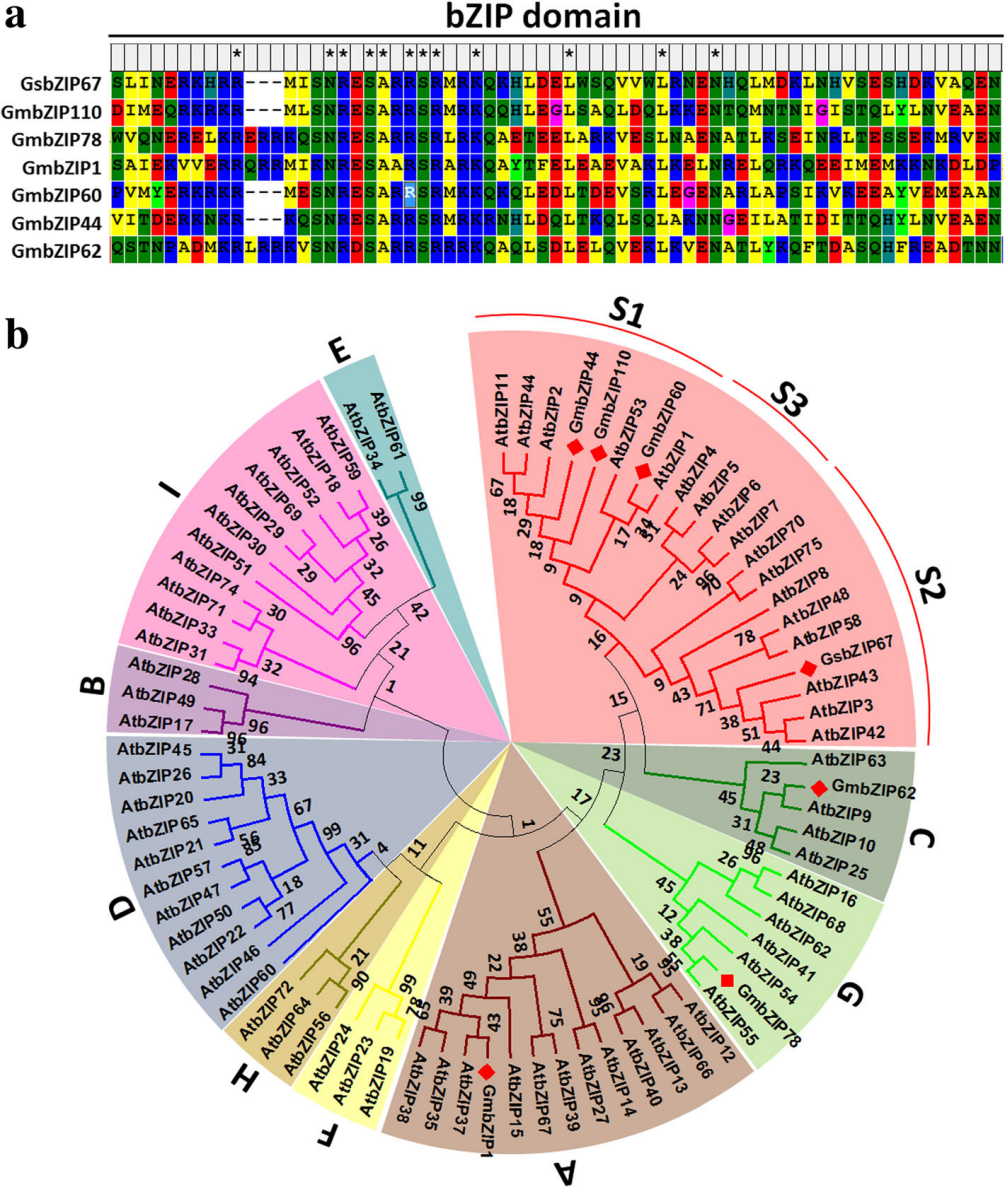
Multiple alignment and phylogenetic analysis of GsbZIP67. **a** Multiple alignment showing the conserved bZIP domain within GsbZIP67. Multiple alignment was performed by using MEGA5.0 with the full-length amino acid sequences, and the results within the bZIP domain were shown. **b** Phylogenetic tree of GsbZIP67 and other bZIP proteins. The phylogenetic tree was constructed by using the neighbor-joining method (with a bootstrap of 1000) with soybean and *Arabidopsis* bZIPs

As shown in Fig. 1a, GsbZIP67 shared high sequence identity within the bZIP domain with other characterized stress-related GmbZIPs, including GmbZIP110 [10], GmbZIP1 [12], GmbZIP60 [22] and GmbZIP44/62/78 [11]. Previous studies have shown that *Arabidopsis* bZIP family was divided into 10 groups (group A-I, S) [3]. The neighbor-joining phylogenetic tree revealed that GsbZIP67, together with GmbZIP44/60/110, was clustered into group S (Fig. 1b). According to a previous report [4], group S was further divided into three clusters (group S1, S2 and S3). As shown in Fig. 1b, GmbZIP44/60/110 belonged to group S1, while GsbZIP67 belonged to group S2.

### GsbZIP67 is a nuclear-localized bZIP TF with transactivation activity

**Fig. 2:**
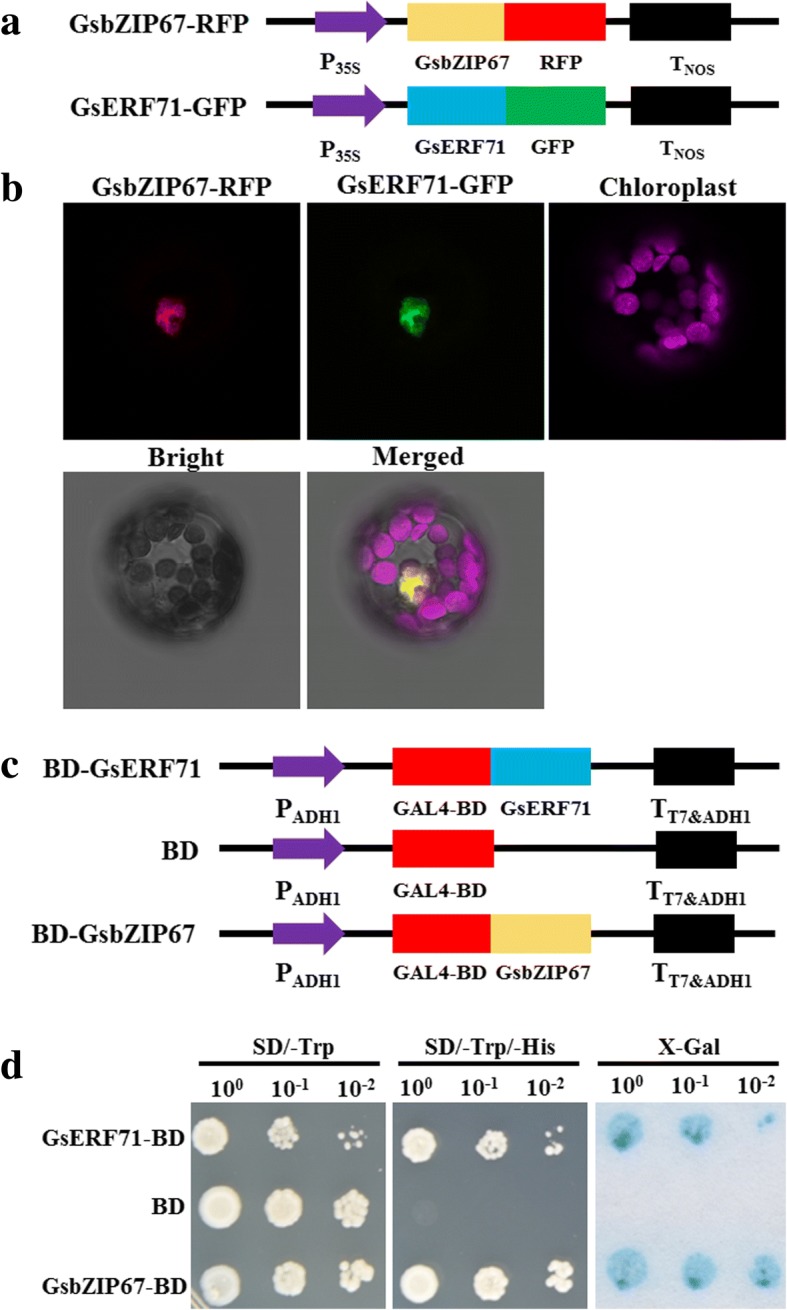
Subcellular localization and transcriptional activation of GsbZIP67. **a** Structures showing the constructs for GsbZIP67 subcellular localization assays. **b** The nuclear localization of GsbZIP67-RFP protein in *Arabidopsis* protoplasts. GsERF71-GFP was used as a nuclear marker. **c** Structures showing the constructs for GsbZIP67 transactivation assays. **d** The transcriptional activation assays of GsbZIP67 in yeast. The BD and BD-GsERF71 were used as negative and positive controls, respectively

Previous studies have shown the nuclear, cytoplasmic [6] and endoplasmic reticulum [23] localization of bZIP TFs. Hence, in this study, to determine the subcellular of GsbZIP67 in plant cells, it was translationally fused with the RFP (Fig. 2a), and co-transformed into *Arabidopsis* protoplasts with GsERF71-GFP, which was used as a nuclear marker [17]. As shown in Fig. 2b, the transformed protoplasts exhibited overlapping RFP and GFP fluorescence in the nucleus, suggesting that GsbZIP67-RFP and GsERF71-GFP co-localized in the nucleus of *Arabidopsis* protoplasts.

We further investigated the transcriptional activation activity of GsbZIP67 in yeast cells. *GsbZIP67* was cloned into the pGBKT7 vector to express BD-GsbZIP67 (Fig. 2c). The empty pGBKT7 vector was used as a negative control, and BD-GsERF71 [17] was used as a positive control. As shown in Fig. 2d, the yeast cells expressing BD-GsbZIP67 and BD-GsERF71 grew well on SD/−Trp-His selective media, and turned blue in the β-galactosidase activity assays, while the BD-containing yeasts could not grow on SD/−Trp-His, and showed no β-galactosidase activity. This finding suggests that GsbZIP67 acts as a transcriptional activator in yeasts.

### The expression profiles of *GsbZIP67* under bicarbonate alkaline stress and in different tissues

**Fig. 3:**
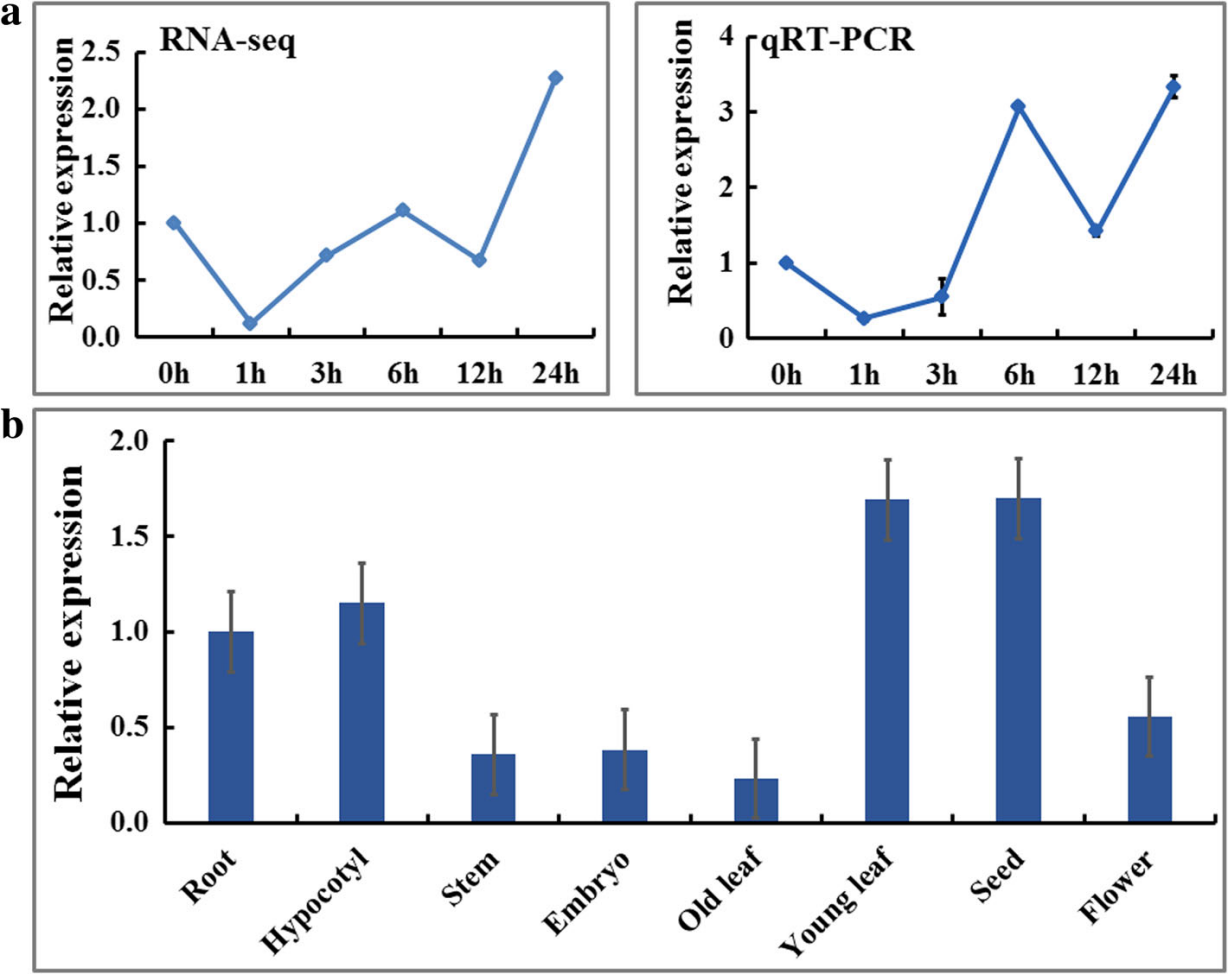
Expression profiles of *GsbZIP67* under bicarbonate alkaline stress and in different tissues. **a** RNA-seq data and qRT-PCR results showing the expression profile of *GsbZIP67* under bicarbonate alkaline stress. The expression level at 0 h was set as 1. **b** QRT-PCR results showing the tissue expression pattern of *GsbZIP67* in wild soybean. The expression level in root was set as 1. *GAPDH* was used as an internal control. Data are means ± SE (*n* = 3)

To verify the RNA-seq data, we analyzed the expression pattern of *GsbZIP67* under 50 mM NaHCO_3_ treatment through quantitative real-time PCR (qRT-PCR) analysis. As shown in Fig. 3a, after NaHCO_3_ treatment, the transcript abundance of *GsbZIP67* declined to the minimum at 1 h, and then started to climb. The expression pattern of *GsbZIP67* revealed by qRT-PCR was consistent with the RNA-seq data, and confirmed that *GsbZIP67* expression is responsive to bicarbonate alkaline stress, implying its potential involvement in plant responses to bicarbonate alkaline stress.

We further investigated the expression profiles of *GsbZIP67* in different tissues, including old leaf, young leaf, root, stem, hypocotyl, embryo, flower and seed (Fig. 3b). Our qRT-PCR results showed that *GsbZIP67* was expressed in different tissues. Among the eight tissues detected in this study, *GsbZIP67* displayed the highest expression level in young leaf and seed, while the lowest level in old leaf.

### Overexpression of *GsbZIP67* in alfalfa confers enhanced bicarbonate alkaline tolerance

To investigate the effect of *GsbZIP67* on bicarbonate stress tolerance, we overexpressed it in *M. savita*, which belongs to the same *Leguminosae* with soybean, but is easier for transformation. To this end, *GsbZIP67* was cloned into the pCAMBIA330035Su vector [24] (Additional file 1: Figure S1) and transformed into alfalfa via *Agrobacterium tumefaciens*-mediated method. The integration of *GsbZIP67* in alfalfa genome was confirmed by southern blot analysis (Additional file 1: Figure S1). The constitutive expression of *GsbZIP67* was further verified through semi-quantitative RT-PCR assays (Additional file 1: Figure S1).

**Fig. 4:**
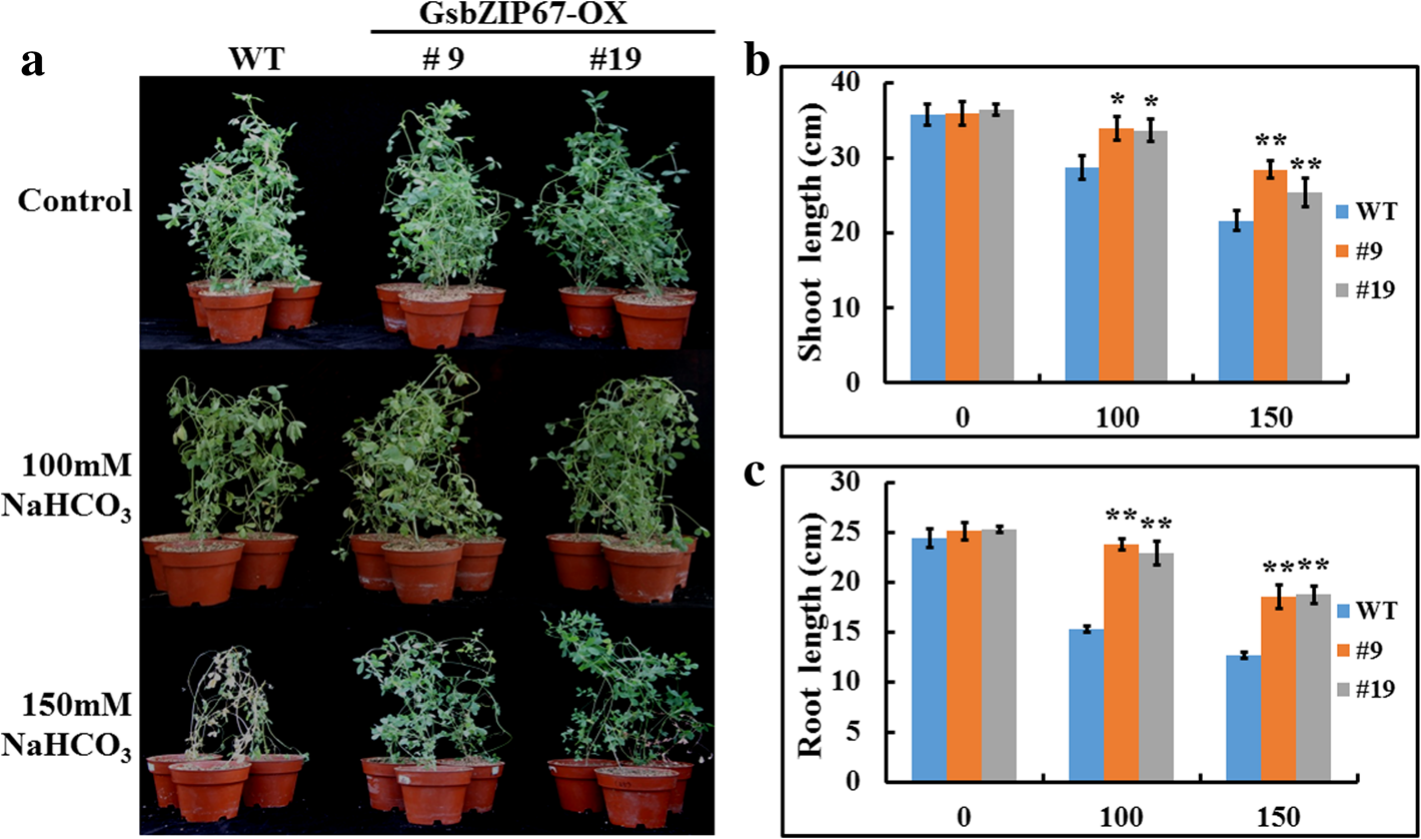
Overexpression of *GsbZIP67* in alfalfa promoted plant growth under bicarbonate stress. Phenotype **(a)**, shoot length **(b)**, and root length **(c)** of the WT and *GsbZIP67* OX lines under both normal conditions and bicarbonate treatment. The 4-week-old WT and OX plants were irrigated with 1/4 Hoagland solution containing either 0, or 100, or 150 mM NaHCO_3_ every 2 days for 14 days. Data are means ± SE (*n* ≥ 10). *P < 0.05, **P < 0.01; Student t-test

**Fig. 5:**
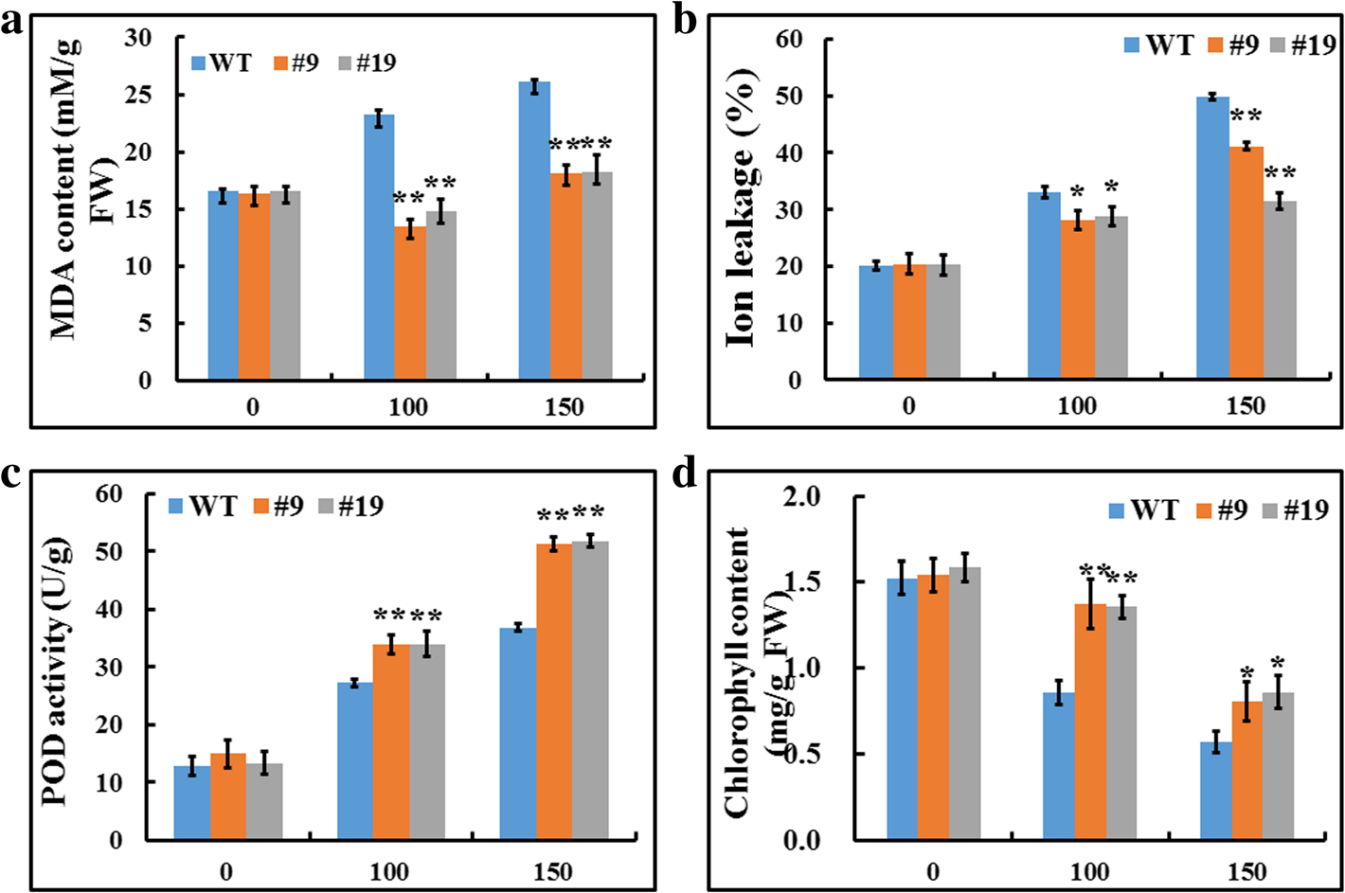
Changes in the physiological indices of *GsbZIP67* transgenic alfalfa under bicarbonate alkaline stress. The MDA content (**a**), ion leakage (**b**), POD activity (**c**) and chlorophyll content (**d**) of the WT and *GsbZIP67* OX lines under both normal conditions and bicarbonate treatment. Data are means ± SE (*n* ≥ 10). *P < 0.05, **P < 0.01; Student t-test

In order to evaluate whether *GsbZIP67* overexpression affects the bicarbonate tolerance, the wild type (WT) and overexpression (OX) alfalfa plants were treated with either 0, or 100, or 150 mM NaHCO_3_ for 14 days. As shown in Fig. 4a, under stress treatment, the OX lines showed much better growth than WT. The quantitative data revealed that the shoot and root length of OX lines were significantly higher than that of WT under NaHCO_3_ treatment (Fig. 4b, c). Quantification of the physiological indices showed that compared with WT, the OX lines exhibited less malon dialdehyde (MDA) accumulation (Fig. 5a) and lower ion leakage (Fig. 5b), while displayed higher peroxisome (POD) activity (Fig. 5c) and chlorophyll content (Fig. 5d) under bicarbonate alkaline stress. Taken together, these results strongly suggest that *GsbZIP67* overexpression enhanced the bicarbonate alkaline tolerance of transgenic alfalfa.

### Overexpression of *GsbZIP67* results in increased expression of stress responsive genes

**Fig. 6:**
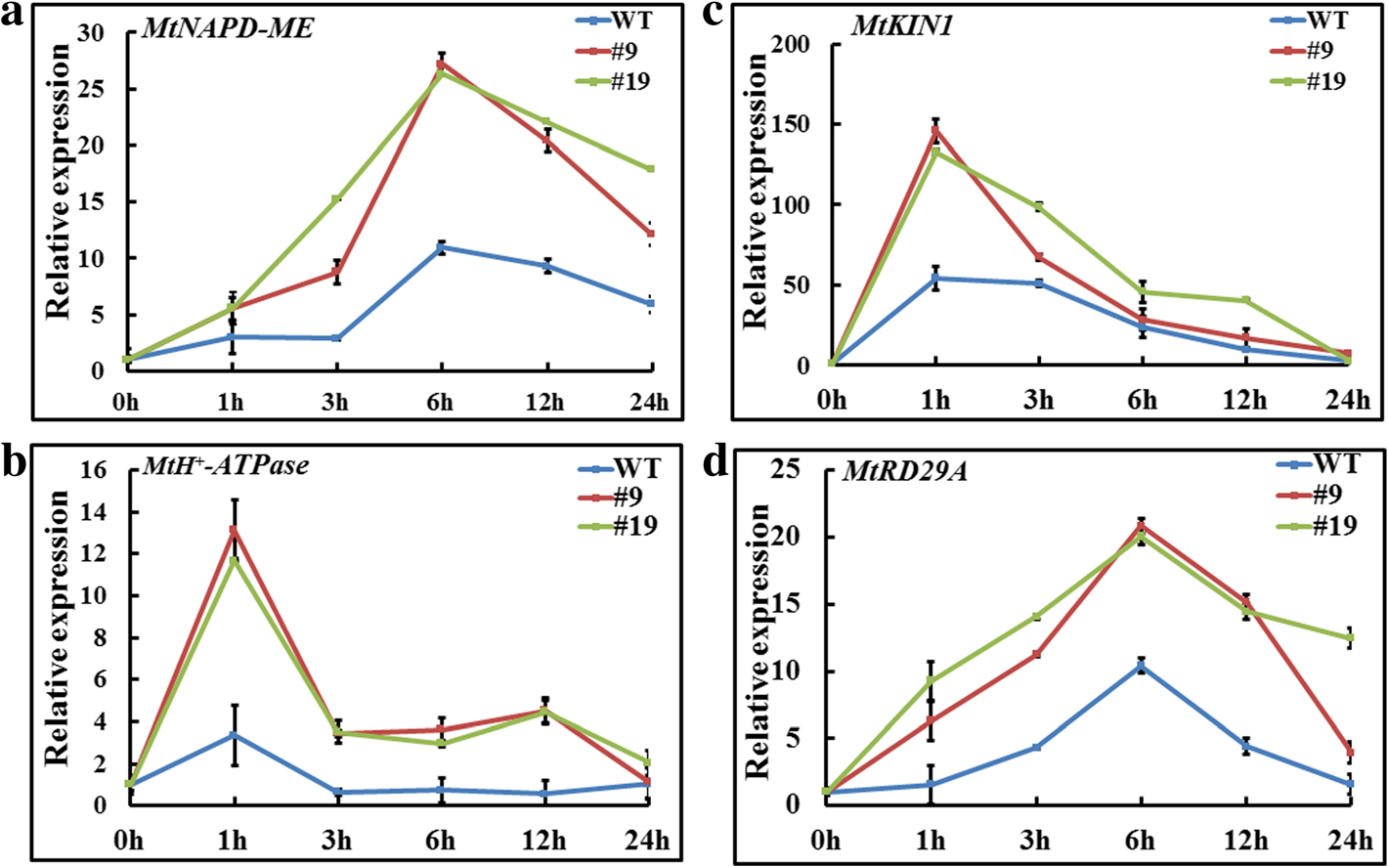
*GsbZIP67* overexpression in alfalfa improved the expression of stress responsive genes. Expression levels of the *MtNAPD-ME* (**a**), *MtH*^*+*^*-Ppase* (**b**), *MtKIN1* (**c**) and *MtRD29A* (**d**) in the WT and *GsbZIP67* OX lines under bicarbonate treatment. *GAPDH* was used as an internal control. The expression level of WT at 0 h was set as 1. Data are means ± SE (*n* = 3)

Previous studies showed that *NADP-ME* [25] and H^+^-pyrophosphatase (H^+^-Ppase) [26] were of great help to maintain the cytosolic pH homeostasis. As determined by the qRT-PCR analysis, the expression of *MtNADP-ME* (Fig. 6a) and *MtH*^*+*^*-Ppase* (Fig. 6b) was induced by NaHCO_3_ treatment in both WT and OX plants. However both of them were up-regulated in the OX lines compared with WT (Fig. 6a, b). Moreover, another two stress responsive marker genes *MtRD29A* and *MtKIN1* were also detected in this study. Our results uncovered that under bicarbonate alkaline stress, the expression levels of *MtRD29A* and *MtKIN1* in the OX lines were obviously higher than that in WT (Fig. 6c, d). Taken together, these findings suggest that overexpression of *GsbZIP67* in alfalfa augmented the transcription of stress responsive genes.

## Discussion

It is widely accepted that bZIP TFs play particularly important roles during a diverse variety of biological processes, such as TGAs (group D) in pathogen defense [27], ABFs (group A) in ABA and abiotic stress tolerance [28], group C/S1 bZIPs in energy homeostasis [5] and HY5/HYH (group H) in light responses [29]. However, most researches about bZIPs were carried out in the reference plants *Arabidopsis* and rice. Little information is reported regarding bZIPs in non-reference plants, especially in legume. In this study, we isolated a bZIP TF from wild soybean *GsbZIP67*, and characterized its positive function in bicarbonate alkaline stress responses.

GsbZIP67 was found to be a group S2 bZIP with nuclear localization and transactivation activity. GsbZIP67 protein contained a well-characterized bZIP domain similar to other stress-related GmbZIPs (Fig. 1a). GsbZIP67 localized in the nucleus of plant cells (Fig. 2b), and displayed transcriptional activation activity in yeasts (Fig. 2d). These findings imply that GsbZIP67 might serve as a transactivator of nuclear gene expression. Furthermore, our studies also unraveled that GsbZIP67 belonged to group S2 (Fig. 1b) [3, 4]. Current researches have uncovered the profound importance of group S1 bZIPs in regulating gene expression by forming heterodimers with group C bZIPs [5, 30, 31]. However, group S2 bZIPs are yet poorly characterized. Even though group S2 bZIPs could not heterodimerize with group C bZIPs, low-affinity interactions were detected inside group S2 bZIPs in *Arabidopsis* [4]. Hence, more experiments are needed to verify whether GsbZIP67 could form homodimers and/or heterodimers with other bZIPs, and whether the dimerization affects its DNA binding affinity and/or transactivation activity.

Numerous studies have unraveled that a large portion of GmbZIPs is differentially expressed under diverse abiotic stresses. A previous genome wide study showed that 75.6% GmbZIPs displayed differential expression after drought and flooding treatments [8]. Experimental evidence was also given to prove the differential expression of some specific GmbZIPs to abiotic stress [9, 11, 12]. A recent study also compared the expression of GmbZIPs in a salt-sensitive cultivated soybean and a salt-tolerant wild soybean [10]. Among them, *GmbZIP110* was up-regulated in both accessions; *GmbZIP78* was up-regulated, while *GmbZIP17* and *GmbZIP91* were down-regulated specifically in the salt-tolerant wild soybean. However, until now, none was reported regarding GmbZIPs responses to bicarbonate alkaline stress. In this study, we showed that *GsbZIP67* expression is firstly decreased, and then increased by bicarbonate alkaline treatment (Fig. 3a). Our previous RNA-seq data [14] also showed that in addition to *GsbZIP67*, expression of 56 GsbZIPs was affected by bicarbonate alkaline treatment (Fold change > 2) (Additional file 2: Figure S2). Some of them displayed similar expression patterns with *GsbZIP67* (firstly decreased and then increased); while others showed opposite (increased and then decreased) expression. Consistently, another RNA-seq study in wild soybean also revealed the differential expressions of bZIP family genes under bicarbonate alkaline stress (90 mM NaHCO_3_) [32]. Transcriptional profiling studies also reported the responsive expressions of bZIPs to bicarbonate alkaline stress in several other plant species [33–35]. In summary, these findings indicate the potential involvement of bZIPs in response to bicarbonate alkaline stress. However, there is no report providing the direct experimental evidence for the genetic and/or functional involvement of bZIPs in bicarbonate alkaline stress.

In this study, we functionally suggested that *GsbZIP67* was a key regulator for plant tolerance to bicarbonate alkaline stress (Figs. 4, 5, 6). Even though previous studies have unraveled the function of GmbZIPs in salt [9–12], drought [11, 12] and cold/freezing [11, 12] tolerance, here our studies, for the first time, reported the novel function of group S2 bZIPs, which are poorly characterized, in regulating bicarbonate alkaline stress responses. Our studies showed that *GsbZIP67* ectopic expression in alfalfa could promote the growth of transgenic plants, and modify the stress-related physiological indices and gene expression (Figs. 4, 5, 6). Notably, *GsbZIP67* overexpression dramatically down-regulated the MDA accumulation (Fig. 5a), but up-regulated the POD activity (Fig. 5c) in transgenic lines compared with WT under bicarbonate alkaline stress. As shown previously, a great increase in MDA accumulation and POD activity is closely related to the responses of alfalfa to bicarbonate alkaline stress [35, 36]. The transcriptional analysis also revealed the differential expression of alfalfa POD family genes under bicarbonate/carbonate alkaline stress [35]. Hence, it is reasonable to speculate that *GsbZIP67* might mediate ROS signaling under bicarbonate alkaline stress.

As alfalfa growth is affected by diverse environmental stresses and salt-alkaline stress always occurs simultaneously, it will be meaningful to determine whether *GsbZIP67* could contribute to increased tolerance to other abiotic stress, such as salt, drought and/or cold tolerance. As *GsbZIP67* is a nuclear transcriptional activator, future studies are needed to identify the direct cis-elements and/or downstream genes targeted by *GsbZIP67* under bicarbonate alkaline stress. Taken together, our results presented in this study provided the genetic evidence of a group S2 bZIP in regulating plant tolerance to bicarbonate alkaline stress. This finding will facilitate to further investigate the regulatory mechanism of bZIPs in bicarbonate alkaline stress.

## Conclusions

In conclusion, the data presented here strongly suggests that *GsbZIP67* acts as a stress-inducible, nuclear-localized group S2 bZIP transcription activator, and positively regulates bicarbonate alkaline tolerance through modifying the stress-related physiological indices and gene expression. This is the first report characterizing the involvement of group S2 bZIPs in bicarbonate alkaline stress. Our results not only broaden the current knowledge of bZIPs in stress tolerance, but also will greatly benefit future research regarding the molecular basis of bZIPs in alkaline stress.

## Methods

### Plant materials and growth conditions

Seeds of *G. soja* (G07256) were obtained from Jilin Academy of Agricultural Sciences, and kept by Plant Bioengineering Laboratory. Wild soybean seeds were treated with 98% H_2_SO_4_ for 10 min to damage seed coat [37, 38], and then allowed to germinate on soggy filter papers in darkness until 2–3 cm buds appeared. The wild soybean seedlings were transferred for hydroponics in 1/4 Hoagland solution. Seeds of *M. sativa* (Zhaodong) was provided by Heilongjiang Academy of Agricultural Science, and used for transformation. The alfalfa plants were grown in pots filled with pearlite:vermiculite (1:1). Both the wild soybean and alfalfa plants were grown in a chamber and/or greenhouse at 24–28 °C and 16/8 h light/dark cycles.

### Isolation and sequence analysis of the *GsbZIP67* gene

The full-length coding region of *GsbZIP67* was PCR-amplified from the wild soybean cDNA templates with gene specific primers (Additional file 3: Table S1). Multiple alignment was performed by using MEGA5.0 with the full-length amino acid sequences with default parameters. The neighbor-joining phylogenetic tree was constructed with soybean and *Arabidopsis* bZIP family proteins, and different bZIP groups were annotated according to previous studies [3].

### Subcellular localization analyses of GsbZIP67 in *Arabidopsis* protoplasts

The *GsbZIP67* CDS region minus the stop codon was cloned into pBSK-RFP to express GsbZIP67-RFP protein. The pBSK-GsERF71-GFP construct [17] was used as a marker for nuclear protein. The GsbZIP67-RFP and GsERF71-GFP constructs were transiently co-expressed in *Arabidopsis* protoplasts as described [39]. RFP/GFP/chlorophyll fluorescence was observed by using a confocal laser-scanning microscope Leica SP8 (Leica, Wetzlar, Germany) with 584/488/488 nm excitation and 607/507/650 nm emission, respectively.

### Transcriptional activation assays of GsbZIP67 in yeast

*GsbZIP67* was cloned into pGBKT7 to produce the BD-GsbZIP67 fusion protein. The empty pGBKT7 and pGBKT7-GsERF71 [17] were used as negative and positive controls, respectively. The pGBKT7, pGBKT7-GsERF71 and pGBKT7-*GsbZIP67* were transformed into yeast strain AH109 through the LiAc-mediated method, respectively. Positive clones were cultured in SD/−Trp liquid medium and a serial of diluted culture (1, 10, and 100) were spotted onto SD/−Trp and SD/−Trp-His medium, respectively, and grown for 3 d at 30 °C. The LacZ activity was assessed by using the β-galactosidase colony-lift filter assay.

### Transformation and molecular identification of *GsbZIP67* transgenic alfalfa

*GsbZIP67* was inserted to the binary vector pCAMBIA330035Su [24]. The recombinant construct was introduced into *A. tumefaciens* strain EHA105, and then transformed into alfalfa by using the cotyledonary node method [40]. The glufosinate-positive plants were firstly verified by PCR analysis using *Bar* specific primers (Additional file 3: Table S1). The PCR-positive plants were further detected by Southern Blot analysis, with *Hind*III and *EcoR*I double-digested genomic DNA. The *GsbZIP67* transcript levels in the transgenic plants were analyzed by semi-quantitative RT-PCR, with *MtGAPDH* (Accession number: Medtr3g085850) as an internal control.

### Phenotypic analysis of *GsbZIP67* transgenic alfalfa under bicarbonate alkaline stresses

The lignified WT and *GsbZIP67* OX alfalfa plants were propagated by stem cuttings [40]. For bicarbonate alkaline stress, the 4-week-old WT and OX plants were irrigated with 1/4 Hoagland solution containing either 0, or 100, or 150 mM NaHCO_3_ every 2 days for 14 days. Photos were taken to show the growth phenotype and the shoot and root length was measured. Samples were harvested for the physiological indices measurement. The MDA content was determined through the thiobarbituric acid assays described previously [41]. The relative membrane permeability was measured using a conductivity meter as described [42]. The POD activity was detected following the method described previously [43]. The total chlorophyll content was determined in 80% (*v*/v) acetone extract by measuring the absorbance at 663 and 645 nm.

### qRT-PCR analyses

To investigate the expression patterns of *GsbZIP67* in wild soybean, the 4-week-old seedlings were treated with 1/4 Hoagland solution containing 50 mM NaHCO_3_. To analyze the expression of stress responsive genes in transgenic alfalfa, the 4-week-old alfalfa plants treated with 1/4 Hoagland solution containing 150 mM NaHCO_3_. Samples were harvested at 0, 1, 3, 6, 12 and 24 h, respectively, and stored at − 80 °C freezer. Total RNA was extracted and subjected to cDNA synthesis as described previously [40]. The cDNA was used for qRT-PCR assays with *GAPDH* in wild soybean (DQ355800) and alfalfa (Medtr3g085850), respectively, as the reference genes, expression of which is not affected by bicarbonate alkaline stress. Expression levels were calculated by using the 2^−ΔΔCT^ method. The expression levels in wild soybean and WT alfalfa at 0 h was set as 1. The gene specific primers used in this study were listed in Additional file 3: Table S1.

## Additional files

Additional file 1: **Figure S1.** Molecular identification of *GsbZIP67* overexpression transgenic alfalfa lines. (TIF 114 kb)

Additional file 2: **Figure S2.** Expression profiles of soybean bZIP family genes under bicarbonate alkaline stress based on RNA-seq data. (PDF 152 kb)

Additional file 3: **Table S1.** Primers used in this study. (DOCX 16 kb)

## Abbreviations

bZIP: Basic leucine zipper;
H^+^-Ppase: H^+^-pyrophosphatase
MDA: Malon dialdehyde
OX: Overexpression
POD: Peroxisome
qRT-PCR: Quantitative real-time PCR
TF: Transcription factor
WT: Wild type
