# Correction to: A *Glycine soja* group S2 bZIP transcription factor *GsbZIP67* conferred bicarbonate alkaline tolerance in *Medicago sativa*

**DOI:** 10.1186/s12870-018-1580-2

**Published:** 2019-01-14

**Authors:** Shengyang Wu, Pinghui Zhu, Bowei Jia, Junkai Yang, Yang Shen, Xiaoxi Cai, Xiaoli Sun, Yanming Zhu, Mingzhe Sun

**Affiliations:** 1 Plant Bioengineering Laboratory, Northeast Agricultural University, Harbin, 150030 People’s Republic of China; 2 Crop Stress Molecular Biology Laboratory, Heilongjiang Bayi Agricultural University, Daqing, 163319 People’s Republic of China

**Correction to: BMC Plant Biology**

**https://doi.org/10.1186/s12870-018-1466-3**

Following publication of the original article [1], the author reported that their given name was misspelled. The details are as follows:

**Incorrect name in the original article:**

Pinhui Zhu

**Correct name:**

Pinghui Zhu

The original article has been corrected.
