# Supplementary figures and images for: A Glycine soja group S2 bZIP transcription factor GsbZIP67 conferred bicarbonate alkaline tolerance in Medicago sativa
Source: BMC Plant Biol. 2018 Oct 13;18:234. doi: 10.1186/s12870-018-1466-3 (PMC6186066; doi:10.1186/s12870-018-1466-3)

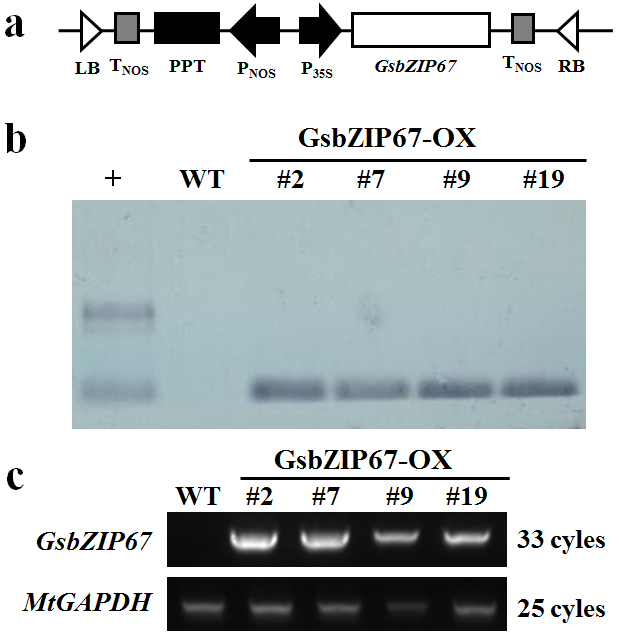

Supplement: Supplementary file 1 — Figure S1. Molecular identification of GsbZIP67 overexpression transgenic alfalfa lines. (TIF 114 kb) [file 12870_2018_1466_MOESM1_ESM.tif]

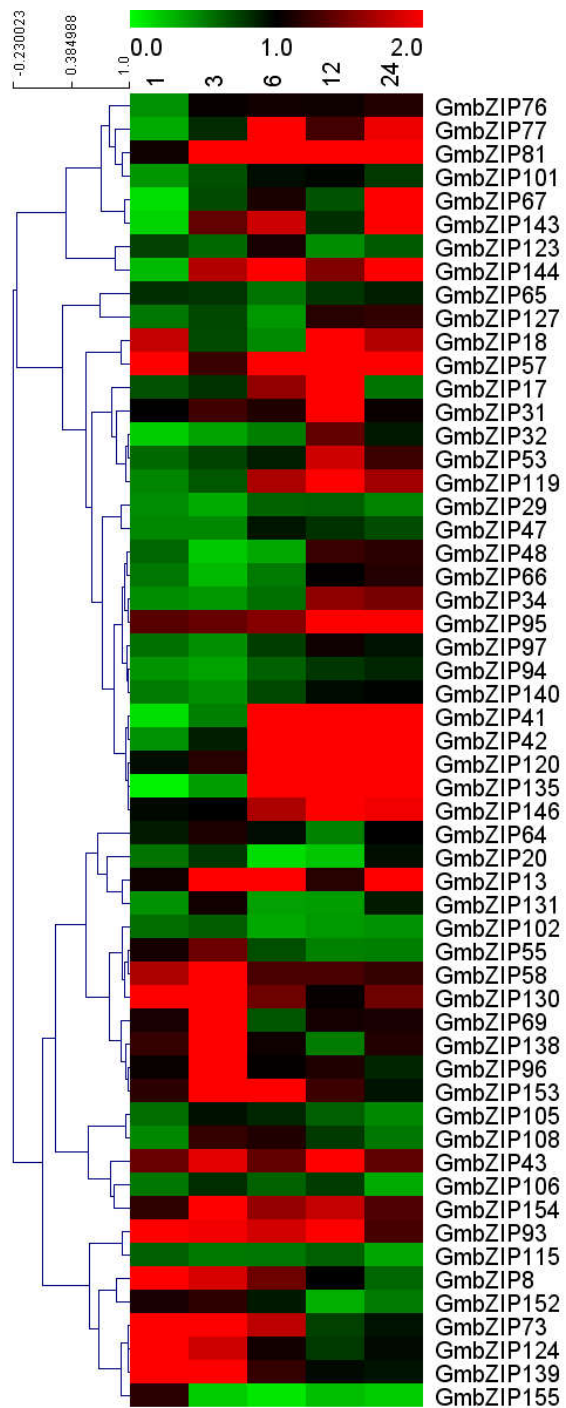

Supplement: Supplementary file 2 — Figure S2. Expression profiles of soybean bZIP family genes under bicarbonate alkaline stress based on RNA-seq data. (PDF 152 kb) [file 12870_2018_1466_MOESM2_ESM.pdf]
