# Supplementary material for: A Glycine soja group S2 bZIP transcription factor GsbZIP67 conferred bicarbonate alkaline tolerance in Medicago sativa
Source: BMC Plant Biol. 2018 Oct 13;18:234. doi: 10.1186/s12870-018-1466-3 (PMC6186066; doi:10.1186/s12870-018-1466-3)
Supplement: Supplementary file 3 — Table S1. Primers used in this study. (DOCX 16 kb) [file 12870_2018_1466_MOESM3_ESM.docx]

| Gene name | Primer sequence (5’-3’) |  |
| --- | --- | --- |
| *GsbZIP67* | Forward: GGCTTAAUATGCAGGCCAGGGAG | For pCAMBIA330035Su |
|  | Reverse: GGTTTAAUTCAACCAAGCAGGTCCA |  |
| *GsbZIP67* | Forward: TTACTCCCTCCAGACCCTTGT  Reverse: AGGTTGAGTTGCTGCTGATGC | For qRT-PCR |
| *GsbZIP67* | Forward: GGAATTCCATATGCAGGCCAGGGAGATC  Reverse: CGGAATTCTCAACCAAGCAGGTCCAAAGAGTC | For pGBKT7 constructs |
| *GsbZIP67* | Forward: GAAGATCTGCAGGCCAGGGAGATCAC  Reverse: GGACTAGTACCAAGCAGGTCCAAAGAGTCTG | For pBSK-RFP |
| *Bar* | Forward: TGCACCATCGTCAACCACTACATCG  Reverse: CCAGCTGCCAGAAACCCACGTCATG |  |
| *GsGAPDH* | Forward: GACTGGTATGGCATTCCGTGT  Reverse: GCCCTCTGATTCCTCCTTGA | For qRT-PCR |
| *MtGAPDH* | Forward: GTGGTGCCAAGAAGGTTGTTAT  Reverse: CTGGGAATGATGTTGAAGGAAG | For qRT-PCR |
| *MtH^+^-ATPase* | Forward: GGCAGCCCTCTACCTACAAGTC  Reverse: AGCAATCATAAAAGCACCCAAT | For qRT-PCR |
| *MtNAPD-ME* | Forward: TAGGTGGAGTTCGTCCTTCAGC  Reverse: AGGTCATAGTATTCCTTCCCAGTTG | For qRT-PCR |
| *MtKIN1* | Forward: AACAAGAATGCCTTCCAAGC  Reverse: CGCATCCGATACACTCTTTCC | For qRT-PCR |
| *MtRD29A* | Forward: ATGATGACGAGCTAGAACCTGAA  Reverse: GTAATCGGAAGACACGACAGG | For qRT-PCR |

**Table S1** Primers used in this study
